# Lifecycle Assessment and Multi-Parameter Optimization of Lightweight Cement Mortar with Nano Additives

**DOI:** 10.3390/ma17174434

**Published:** 2024-09-09

**Authors:** Yiying Du, Aleksandrs Korjakins, Maris Sinka, Ina Pundienė

**Affiliations:** 1 Laboratory of Concrete Technology, Institute of Building Materials, Vilnius Gediminas Technical University, Linkmenu Str. 28, LT-08217 Vilnius, Lithuania; yiying.du@vilniustech.lt (Y.D.); ina.pundiene@gmail.com (I.P.); 2 Institute of Sustainable Building Materials and Engineering Systems, Faculty of Civil and Mechanical Engineering, Riga Technical University, Kipsalas St. 6A, LV-1048 Riga, Latvia; maris.sinka@rtu.lv

**Keywords:** compressive strength, economic analysis, environmental impacts, fly ash cenospheres, flexural strength, TOPSIS method

## Abstract

With the growing global concerns regarding sustainable development in the building and construction industries, concentration only on the engineering properties of building materials can no longer meet the requirements. Although some studies have been implemented based on the lifecycle assessment of lightweight cement-based materials, very few attempts have been made pertaining to multi-criteria optimization, especially when fly ash cenospheres are used as lightweight aggregates and nano additives are incorporated as modifying admixtures. This investigation utilized cenospheres as fine aggregates to produce green, sustainable, lightweight cement mortar. Multi-walled carbon nanotubes at 0.05, 0.15, and 0.45% were binarily added, together with 0.2, 0.6, and 1.0% of nano silica to improve the mechanical performance. Strength tests were conducted to measure the flexural and compressive behaviors, combined with a cradle-to-gate lifecycle assessment and direct cost analysis to assess the environmental and economic viability. Integrated indexes and the TOPSIS method were adopted to systematically evaluate the mortar mixes and determine the optimal mix. The outcomes show that nano additives worked synergically to enhance the mechanical properties of the mortars. The utilization of cenospheres effectively reduced environmental impacts and improved economic feasibility. Nano additives significantly affected the sustainability and economic viability; in particular, the utilization of multi-walled carbon nanotubes increased the material costs. To minimize the impact of the price of multi-walled carbon nanotubes, it is proposed to binarily use less expensive nano silica. In the multi-parameter optimization, the mix with 0.05% multi-walled carbon nanotubes and 0.02% nano silica was recommended to be the optimal mix.

## 1. Introduction

As elemental materials for buildings and structures, the demand for cement-based materials has increased over the years due to the rapid urbanization and ongoing development of the construction sector [1,2]. This has led to significant environmental pressures. The fabrication of ordinary Portland cement (OPC) is highly energy-intensive and responsible for a large quantity of greenhouse gas emissions. According to Abdalla et al. [3], as well as Oyebisi and Alomayri [4], a total amount of approximately 5 to 6 billion tons of OPC is manufactured globally every year, accounting for the emission of greenhouse gases at the rate of 25 to 30 billion tons per annum and consuming 14% of the global industrial energy. Meanwhile, approximately 48.3 billion tons of aggregates were consumed per year to manufacture concrete [5], placing threats on non-renewable natural resources. Mining, operating, and transporting natural aggregates are also connected with considerable energy consumption and carbon dioxide (CO_2_) emissions [6].

Under this background, lightweight concrete (LWC) has gained significant attention and undergone considerable development. According to the EN 206-1:2000 standard [7], LWC is usually classed as concrete materials with a density ranging from 800 to 2000 kg/m^3^. The reduction in density benefits the decrease in material consumption, energy demand, and construction expenditure [8,9]. Combining low-cost and zero-cost industrial and agricultural wastes as lightweight aggregates (LWAs) can reduce the environmental pressure of waste disposal [10,11]. Compared to traditional LWAs, such as waste glass and expanded polystyrene beads, as an industrial by-product of fly ash cenospheres (FACs) are one of the most valuable waste aggregates and are preferred, also owing to their outstanding engineering properties [12]. Their application in producing LWC is considered a sustainable move.

Notwithstanding, the decreased density of LWC leads to a reduction in the mechanical strength. To enhance the engineering performance, investigations have been carried out on the modification of FAC-incorporated lightweight cement composites (LWCCs) via additives such as polyvinyl alcohol fibers, polyethylene fibers, and silica fume [13,14,15,16,17]. Along with the achievements of nanomaterials on the reinforcement of cement-based materials, nano additives have been introduced to LWCCs. Among various categories of nanomaterials, nano silica (NS) and multi-walled carbon nanotubes (MWCNTs) have drawn broad interest. The filling and nucleation effects of NS and MWCNTs enable them to fill the micro-voids in the cement matrix and act as nucleating sites for the hydration reactions, which effectively improves the pore structure and facilitates the hydration degree of the materials [18,19,20]. NS can participate in the pozzolanic reactions, consuming calcium hydroxide (CH) and generating calcium silicate hydrate (CSH) gel, ameliorating the microstructure of cement composites [21]. MWCNTs have the effect as fibers of bridging the micro-cracks in the cement matrix and preventing their propagation [22]. Although there are some studies implemented to identify the influence of nano additives on LWCCs [23,24,25,26], they mainly concentrate on single effects and solely focus on the mechanical performance as well as the micro-mechanisms behind it.

Following global attempts to minimize environmental threats and control production costs, only concentrating on the study of mechanical performance can no longer fulfil the demands of sustainable construction. It is also necessary to include environmental and economic assessments when new building materials are developed. Lifecycle assessment (LCA) is a useful tool to evaluate and compare the environmental impacts of various materials, products, processes, and systems over the whole lifecycle, including the extraction of raw materials, fabrication, usage, and maintenance of the products, as well as the disposal and recycling procedures at the end life of the object [27]. There are four specified steps in the analysis of LCA, including the interpretation of the assessment goal and scope, input of lifecycle inventory, evaluation of environmental impacts, and the description of acquired results [28]. LCA benefits the evaluation of different options of materials and assists in controlling the energy demand, greenhouse gas emissions, and resource consumption by providing a thorough analysis of the system [29,30]. Cost analysis is usually conducted together with LCA to evaluate the economic viability of the materials. Besides the lifecycle costing, direct cost analysis is also applied in the investigations [4,24,31].

Multi-response assessment and optimization is a useful mathematical tool for comparing and determining the optimal mix. Combining the mechanical properties with environmental and economic impacts, some studies have proposed the idea of integrated indexes to assess the comprehensive behavior of cement composites. Ma et al. [32] calculated the cost index and embodied CO_2_ index based on the total cost, CO_2_ emissions, and compressive strength to analyze the cost efficiency and environmental sustainability per unit strength of geopolymer mortar. Adesina and Das [33] introduced the embodied carbon index, embodied energy index, and cost index by dividing the sustainability and cost criteria of each mix with its corresponding tensile ratio. The technique for order preference by similarity to ideal solution (TOPSIS) method is also a widely adopted multi-criteria decision-making (MCDM) system for the optimization of the mix design based on diverse parameters. It requires only simple algorithms [34]. It starts with establishing the decision matrix, then, by calculating the difference between each alternative and the positive or negative ideal solution, the relative closeness coefficient (RCC) is obtained, which is the basis for deciding the optimal system [35]. Many researchers [31,34,36,37,38,39] have applied this approach in their investigations of cement-based materials, such as polymer blended concrete, recycled aggregate concrete, concrete with waste glass, tire aggregates, FACs, etc.

In lightweight cement-based materials, it is expected that environmental and economic analysis are included in the investigation, especially when recycled aggregates (industrial and agricultural wastes) are utilized. For instance, Zhou et al. [40] performed ReCiPe midpoint method-based LCA on engineered LWCCs with limestone calcined clay cement and FACs. Tiong et al. [41] introduced eggshell powder into lightweight foamed concrete, and based on the results from the LCA, the environmental burdens in climate change, eutrophication, acidification, ozone layer depletion, fossil fuel, and photochemical oxidation were successfully reduced by 6.6% to 9.9%. Kumar [42] fabricated a green LWC and conducted LCA as well as the cost analysis, reporting that the addition of micro-fine stone sludge contributed a decline in both production costs and environmental threats. Guo et al. [43] developed an ultra-high-performance lightweight concrete incorporated with hollow glass microspheres, expanded glass aggregate, and polyethylene fibers. They carried out LCA and concluded that 20% and 16% of reductions were observed in carbon footprint and embodied energy, respectively. Napolano et al. [44] conducted LCA on recycled LWAs from industrial wastes. After the comparison with LWAs from natural clay, they reported that using recycled LWAs can effectively lower the environmental burden of LWC. Other researchers also implemented LCA and cost analysis on LWAs or LWCCs with waste materials such as ashes from municipal solid waste incineration, high-volume quarry wastes, ornamental stone processing waste, industrial solid waste, recycled plastic, etc. [45,46,47,48,49].

However, less literature has been reported regarding the multi-response optimization of lightweight cement-based materials. Only Sldozian et al. [50] adopted the TOPSIS method to determine the optimal mix of LWC modified by MWCNTs based on three parameters (the content of MWCNTs, compressive strength, and flexural strength). To-on et al. [51] performed a multi-response optimization on LWC blocks with sugarcane bagasse ash via the TOPSIS linear programming model developed based on the original TOPSIS and response surface methods. They selected three criteria: compressive strength, dry density, and water absorption. Petrillo et al. [52] conducted a comprehensive study combining LCA, cost analysis, and multi-criteria optimization on several artificial LWAs recycled from industrial wastes. Ghazy et al. [53,54] combined the factors of LWA type and dosage and foam agent content and carried out a series of multi-objective optimization on LWC to determine the best mix.

Although the sustainability of lightweight materials is frequently mentioned in investigations, quantitative analysis to address their eco-efficiency is still required; in particular, systematic ones covering the environmental assessment, economic analysis, and multi-response optimization of these materials are still lacking. FACs and nano additives are increasingly used in producing lightweight cement-based materials, which highlights the significance of discussing their influence on not merely the engineering properties but also the sustainability and economic viability of the materials. Therefore, to meet the demand for sustainable production and construction in the building industry, in this study a sustainable green lightweight cement mortar (LWCM) was developed by adding 73.3% of FACs as lightweight fine aggregates and MWCNTs and NS at various dosages as modifying admixtures. The study aims to shed lights on the binary effects of nano additives on the evolution of mechanical properties and reveal the environmental and economic impacts, providing a comprehensive reference on the development of lightweight materials for future research in this field. Both flexural and compressive strength were tested with a cradle-to-gate LCA, in conjunction with a direct cost analysis. Based on the mechanical strength and environmental and economic indicators, a multi-parameter evaluation of LWCM mixes was performed via the sustainability index (SI), economic index (EI), and TOPSIS method to determine the optimal mix. Measured recommendations for optimal dosages of nano additives in building materials are presented.

## 2. Materials and Methods

### 2.1. Materials

**Figure 1:**
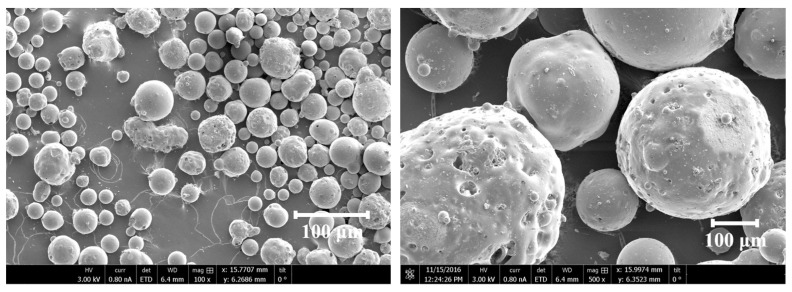
Micrographs of FAC.

OPC from Schwenk, conforming to EN 197-1: 2011 [55], was used. The category of the OPC is CEM 1 42.5 N, and the average bulk density is from 0.9 to 1.5 g/cm^3^. The 28-day standard strength varies from 42.5 to 62.5 MPa. Natural river sand (NRS) from Žvyro Karjerai (Trakai, Lithuania) was used as natural fine aggregates to fabricate natural aggregate cement mortar (NACM) as a control sample. FACs were utilized as lightweight fine aggregates for casting lightweight cement mortar (LWCM). The bulk density of FACs ranges between 0.37 and 0.40 g/cc, and the pH value is 7–8. The shape of FACs, as shown in Figure 1, is a hollow sphere with a particle diameter in the range of 40 to 300 µm and the surface being smooth or rough. Table 1 demonstrates the material properties of OPC and FAC. To modify the mechanical behaviors of LWCM, the MWCNTs from Arkema (Colombes, France) and NS from Aldrich Chemistry (St. Louis, MO, USA) in the powder were added together. The category of NS belongs to silica gel produced from sodium silicate. The synthesis method of MWCNTs is chemical vapor deposition (CVD). The diameter of MWCNTs ranges from 6 to 15 nm. The length varies between 4 and 13 nm, and the specific area is 233 m^2^/g. Melment F 10, manufactured by BASF Corporation (Ludwigshafen, Germany), was used as the surfactant to acquire better-dispersed nanosuspension. The color of Melment F10 is white, and its bulk density varies from 500 to 800 kg/cm^3^. Tap water was adopted during the preparation of the mortar samples. The fly ash cenospheres were produced at the city Zuyev’s thermal power plant in Donetsk region, Ukraina. Their composition was chemically determined to be 53.8 ± 0.5 wt % SiO_2_, 40.7 ± 0.7 wt % Al_2_O_3_, 1.4 ± 0.2 wt % CaO, and 1.0 ± 0.2 wt % Fe_2_O_3_, plus smaller amounts (below 1 wt %) of MgO, Na_2_O, and K_2_O. Bulk density of the particles prior to coating was 0.39 ± 0.006 g/cm^3^ based on repeated Scott volumeter measurements according to ISO 3923-2-81. 

### 2.2. Mix Design

In Table 2, the mix ratio and the corresponding quantity of each raw material required for the preparation of 1 m^3^ cement mortar are specified in detail. Thirteen LWCMs were studied, plus one NACM as a referencing sample. Seventy-three percent of fine aggregates by cement weight were added in the investigation, and two categories of fine aggregates were involved (natural aggregate NRS and LWA FACs). An amount of 0.5% of Melment F10 by cement mass was added to facilitate the dispersion of nano additives. The water/cement (w/c) ratio was 56% to guarantee the workability of cement mortar and the quality of nano-suspension. Due to the hollow and spherical shape of FACs, which can result in air incorporation in the cement mortar during the process of casting samples, the improvement of the w/c ratio can benefit the consistency and workability of the samples [56]. Meanwhile, with more water in the system, nanomaterials, especially MWCNTs, can be better dispersed [57]. The content of nano additives was determined based on the relevant literature [58,59,60,61,62]. The dosage of MWCNTs varied in a range of 0.00, 0.05, 0.15, and 0.45%, and the amount of NS was 0.00, 0.20, 0.60, and 1.00% by cement weight. The abbreviation N in Table 2 indicates NACM, L refers to LWCM, T means the content of MWCNTs, and S illustrates the dosage of NS. For instance, mix ID NT0S0 refers to NACM samples with NRS as fine aggregates, and mix LT15S2 indicates LWCM samples containing 0.15% MWCNTs and 0.2% NS. Note that the proportion of raw materials is based on the cement weight and the material demand is for 1 m^3^ of cement mortar.

### 2.3. Preparation of Samples

To obtain high-quality nanosuspension, in this study the suggested method used in most of the literature [63,64,65] was adopted, with the addition of surfactant and ultrasonic treatment. Initially, Melment F10 was weighed and mixed into one-third of the total amount of water. Then, different dosages of NS and MWCNT powder were added to the solution, which was homogeneously and thoroughly stirred before being subjected to ultrasonic waves. UP50H ultrasonic apparatus from Hielscher Ultrasonics (Teltow, Germany) was used, and the solution was ultrasonicated for 30 min under 30 kHz and 50 W power.

Afterwards, the nanosuspension was mixed with the remaining two-thirds of the water and the solutions were stirred evenly for 2 min. The dry mixture of OPC and FACs was thoroughly stirred for 2 min and then mixed with the solution. After being stirred using an electronic mixer for 3 min, a mini-slump experiment was carried out on a miniature slump cone with 60 mm height and 100 mm bottom diameter to test the workability of the mixture. The outcomes indicated that, by replacing NRS with FACs, the workability of cement mortar significantly decreases, but the addition of nano additives at proper dosages can improve the slump-flowability of LWCM by up to twice the values. Afterwards, the mixture was poured into greased metal prisms from Liming Heavy Industry (Wuxi, China) in three layers and stirred on a vibration table for 2–3 min. The size of the prism molds was 160 mm × 40 mm × 40 mm. All molds were covered by a plastic film at room temperature, and after 24 h, the samples were de-molded, labelled, and immediately placed in a curing tank for 28 days under room temperature. For each mix, 5 samples were prepared. The average density of LWCM was approximately 1120 kg/m^3^, around half that of NACM (1970 kg/m^3^).

### 2.4. Flexural and Compressive Strength

After 28 days, the flexural strength was examined on a WDW–20 universal testing apparatus (Suzhou Jianzhuo Instrument Technology Co., LTD, Kunshan City, China) with loading at 2 mm/min. Then, the fractured specimens were continuously subjected to compressive tests using a 50–C56G2 strength testing machine from CONTROLS (Milan, Italy).

### 2.5. Environmental Impacts and Lifecycle Assessment

#### 2.5.1. Goal and Scope of Lifecycle Assessment

**Figure 2:**
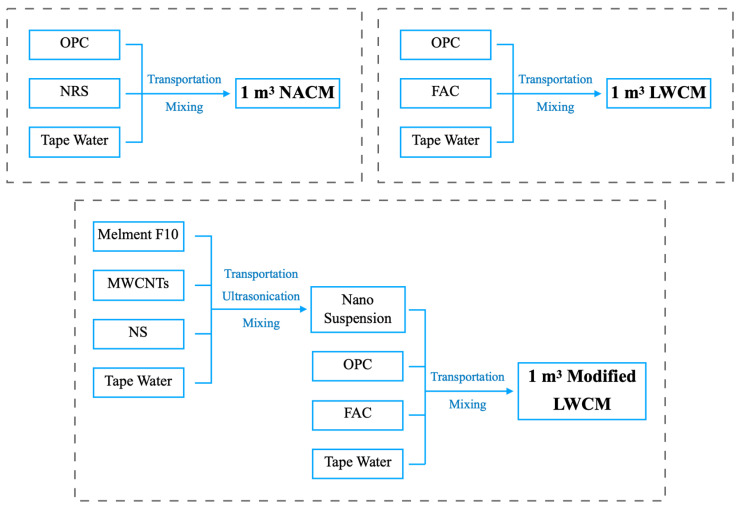
System boundary of LCA.

To evaluate the environmental influence of the fabricated LWCM and compare it to NACM, a cradle-to-gate LCA was carried out, which encompasses all material flows of inputs and outputs from the ground to the industry gate and covers all the processing operations [4]. The functional unit was defined as the manufacture of 1 m^3^ of LWCM and NACM, and the system boundary is illustrated in Figure 2. The production procedure of the mortar product encompassed a variety of steps, which can be specified mainly as follows: (1) extraction of raw materials; (b) transportation of raw materials per unit product to the mixing site; (3) preparation of per unit cement mortar in the mixing plant.

#### 2.5.2. Lifecycle Inventory

The lifecycle inventory (LCI) in the analysis was mainly obtained from the Ecoinvent 3.01 database. Inventory data that was unavailable in the database was adopted as specified in the relevant literature. LCI for the production of NACM and LWCM involved the following: (1) manufacture and use of OPC; (2) extraction and processing of NRS as fine aggregates; (3) use of tap water; (4) production and processing of MWCNTs, NS as well as Melment F10; (5) use of industrial by-product FACs; (6) transportation of materials. The input–output data of OPC, natural fine aggregates, surfactant Melment F10, and water of global scale acquired from the database is shown in Table 3.

The inventory information on NS was obtained from the research conducted by Rose et al. [66] and Mahapatra et al. [67]. For the inventory of MWCNTs, in the previous relevant research, Isaacs et al. [68] compared the environmental impacts and energy consumption of various methods to manufacture single-walled carbon nanotubes (SWCNTs). Khanna et al. [69] investigated the lifecycle of the production process of vapor-grown carbon nanofibers (CNFs). Kushnir and Sandén [70] studied the cumulative energy consumption of different approaches to manufacturing SWCNTs and MWCNTs. Overall, the comprehensive information regarding the LCI of MWCNTs is limited in the existing literature. In this study, the energy consumption data to fabricate MWCNTs via the floating catalyst CVD method with gas benzene as feedstock in the investigation conducted by Kushnir and Sandén [70] were used to estimate the environmental impacts of MWCNTs. Sonication treatment was used to fabricate nanosuspension, the inventory of which was also encompassed into the LCA. In the research performed by Vauchel et al. [71], all the equipment (including the ultrasonication apparatus) was converted into LCI by applying a mass allocation based on the weight, lifespan, and daily usage duration of the equipment. Another approach was proposed by Arvidsson et al. [72], converting the inventory of sonication into electricity input through estimation based on the linear relation between the power required to produce ultrasound and the solvent volume. In this study, the second method was adopted, and the linear approximation of the input electricity was performed based on the experimental data acquired in laboratory settings.

Concerning the LCI of FACs, there are two options to include the input–output data. Xie et al. [73] referenced the LCI of fly ash as the data source for FACs, while Tang et al. [74] introduced 1.5% of the allocation to FACs from the manufacture of fly ash. According to Chen et al. [75] and Zhang et al. [76], inventory allocation is significant for recycled aggregate mortar and directly affects the analysis results. The FACs used in this study also belong to waste by-product; therefore, an allocation of 1.5% of the environmental impacts was applied to FACs from fly ash production.

#### 2.5.3. Impact Assessment

According to Pradhan et al. [28], three main steps are incolved in the procedure of impact assessment: (1) identification of impact categories; (2) classification and specification of LCI to the corresponding impact categories; (3) transformation of impacts into indicators through aggregating LCI. To obtain indicative outcomes associated with CO_2_ emissions and total energy consumption, SimaPro 8.0 software was used to perform an impact assessment and calculate the values of the indicators. As shown in Table 4, global warming potential (GWP) was analyzed based on the IPCC GWP 100a method, and energy requirement was quantified with the cumulative energy demand (CED) method, which is regarded as an important concern in civil engineering.

### 2.6. Economic Assessment

To evaluate the economic impacts of LWCM mixes, the total material consumption (TMC) per unit of cement mortar was cumulated, and the overall expenditure of raw materials (RME) for each mix was calculated. In most of the literature regarding cost analysis, the material price was adopted based on the price provided by local suppliers or the market price from global producers [24,31,77]. Similarly, the material price from the suppliers available worldwide was used to approximate the raw material cost. Table 5 shows the unit price of the raw materials used in this study, converted into Euros (EUR). Note that no economic influence of transportation and material processing was considered during the calculation of material costs, because the transportation expenditures rely on the distance and this investigation aims to compare only the economic impacts associated with raw materials. The indicators and methods to assess the economic impacts are shows in Table 6.

### 2.7. Multi-Criteria Optimization

To further compare and identify the optimal LWCM mix, based on LCA, economic analysis, and mechanical strength, multi-criteria optimization was implemented, taking into account the compressive strength, flexural strength, environmental influence, material consumption, energy demand, and raw material expenditure. Five specimens that had achieved positive results in all the strength tests have been evaluated in this section, including mixes LT5S0, LT15S0, LT5S2, LT15S2, and LT15S6.

Similar to the concepts proposed by Ma et al. [32], Oyebisi et al. [4], Adesina and Das [33], and Refaat et al. [78], for the purpose of assessing the overall sustainable viability and economic influence of modified LWCM, a simplified analysis via SI and EI was conducted using a similar method, with some modifications. SI is an environmental indicator to interpret the environmental impacts by relating the greenhouse gas emissions of different mixes to their 28-day mechanical strength. The values of SI were determined based on the outcomes in compressive strength, flexural strength, GWP, and CED, as calculated using the following Equation (1):

(1) $$SI=\frac{GWP+{CO}_{2i}\times CED}{f_{c}+f_{f}}$$

where:

*SI* = Sustainability index (kg CO_2_ eq/MPa);

*GWP* = Global warming potential (kg CO_2_ eq);

*CED* = Cumulative energy demand (MJ);

*CO*_2*i*_ = CO_2_ intensity of the energy supply, equal to 0.05 kg CO_2_ eq/MJ [4];

*f_c_* = 28-day compressive strength (MPa);

*f_f_* = 28-day flexural strength (MPa).

Also, to compare the economic effects of LWCM mixes, EI was analyzed by connecting the total production costs of each mix to its 28-day compressive and flexural strength. Its value was determined following Equation (2) below:

(2) $$EI=\frac{RME}{f_{c}+f_{f}}$$

where:

*EI* = Economic index (EUR/MPa);

*RME* = Raw material expenditure (EUR);

*f_c_* = 28-day compressive strength (MPa);

*f_f_* = 28-day flexural strength (MPa).

**Figure 3:**
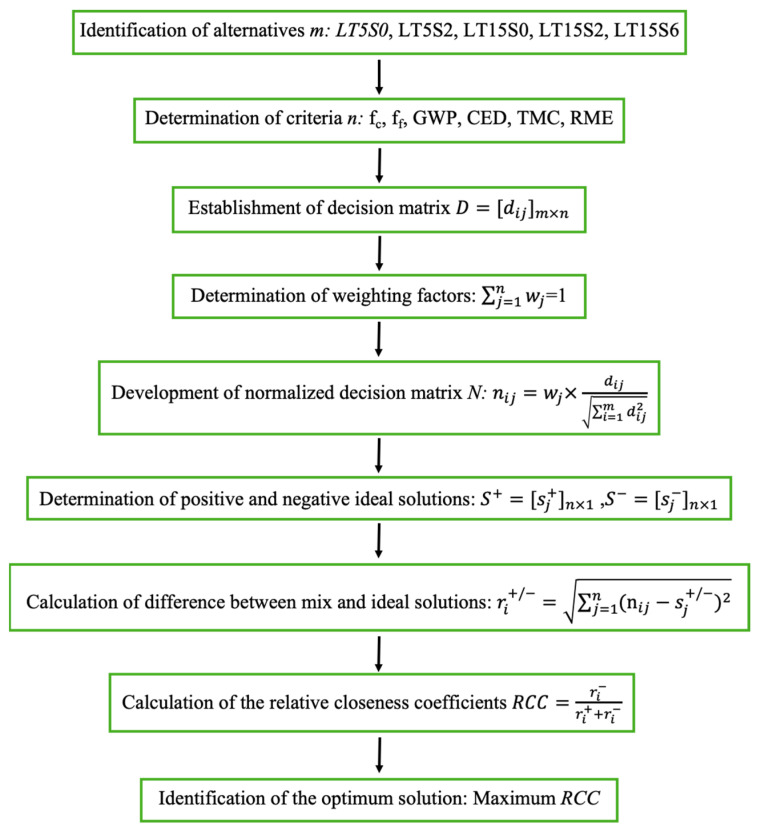
Schematic procedure of the TOPSIS method.

The MCDM method is usually utilized to conduct thorough comparison and optimization according to the comprehensive consideration of a variety of factors that affect the total behavior of the system. Among a diversity of MCDM methods, the TOPSIS method proposed by Hwang and Yoon [79] is an effective evaluation approach and is preferred because it is easier to carry out than other methods and does not require complex mathematical analysis [31,39,80]. As displayed in Figure 3, the first step is to define the target alternatives and the criteria, which in this study encompassed flexural strength, compressive strength, GWP, CED, TMC, and RME. Then, a decision matrix, D, is established combining the alternatives and criteria, which is afterwards weighted and developed into the normalized decision matrix, N. Then, the positive and negative ideal vectors (S^+^ and S^−^) are generated to represent the best and worst solutions, respectively. The relative closeness coefficient (RCC) of each alternative, which is the optimization basis, is determined by the calculation of the difference between each alternative and the positive or negative ideal solutions. At the eventual step, the optimal mix is selected according to the maximal value of RCC. Note that it is better that the criteria for mechanical strength are higher, whereas it is better that the criteria for environmental sustainability and economic feasibility are lower.

Four scenarios of TOPSIS analysis in Table 7 were included in this study based on the different weighting factors. In the first circumstance, the same importance was assigned to all six criteria, which were presumed to possess the same priority. In the second situation, the greater significance was attached to the mechanical strength, with 0.3 for criteria 1 and 2. The third analysis assumed that environmental feasibility held the highest priority and had a weight of 0.3 (criteria 3 and 6), whereas, in the fourth context, the most crucial factor was believed to be the economic viability, and thus criteria 4 and 6 possessed the weight of 0.3.

## 3. Results

### 3.1. Flexural Strength

**Figure 4:**
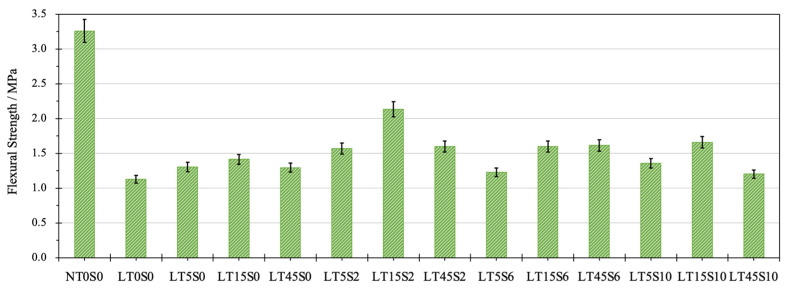
Flexural strength of cement mortar.

The mean values of flexural strength and the corresponding standard deviation of 14 cement mortar mixes are presented in Figure 4. Without the addition of nano additives, the flexural strength of LWCM reached 1.13 MPa, which was 65% less than the flexural strength of NACM containing NRS (3.26 MPa). For every 1% reduction of weight, almost 1.47% loss of flexural strength was witnessed, which demonstrates the significance of enhancing the mechanical characteristics of LWCM via the usage of effective modifiers. In contrast with the unmodified LWCM mix LT0S0, remarkable improvements in flexural strength were observed after MWCNTs and NS were incorporated. The maximal flexural strength of 2.14 MPa, around 65.64% of the strength of NACM, was measured for mix LT15S2, including 0.15% MWCNTs and 0.2% NS, with an increase of 89.38%. However, the density of LT15S2 was 1179.70 kg/m^3^, which was only 59.78% of the NACM density. Mix LT45S10, with 0.45% MWCNTs and 1.0% NS, displayed the lowest enhancement in flexural strength, with only 6.75% of growth to 1.20 MPa. Other dosages of nano additives contributed to the strength development from 10% to 50%.

With the absence of NS, by increasing the dosage of MWCNTs to 0.15%, the flexural strength continued growing to 1.42 MPa. This is due to the positive effect of MWCNTs to bridge the micro-cracks in cement mortars and introduce additional closing against crack propagation [81,82]. Afterwards, the further enhancement of MWCNT content led to a slight drop of strength by 0.12 MPa, which is associated with the agglomeration and flocculation of MWCNTs at an exceeding amount, adversely affecting the strength gain [83]. The synergic effect was observed after the binary addition of NS at proper contents, which further improved the flexural strength. The incorporation of 0.2% NS displayed, on average, better effects than other dosages. When the content of MWCNTs was fixed at 0.05% and 0.15%, compared to the mixes with only MWCNTs, NS addition increased the flexural strength at first by 19.85% and 50% to the peak value, respectively, at 0.2% NS content. This was followed by a decline to the lowest strength value of 1.22 and 1.61 MPa, respectively, at 0.6% of NS, and a slight growth afterwards. The initial strength improvement is related to the ameliorated adhesion between the cement matrix and MWCNTs due to the presence of NS, but with the growing amount of NS particles in the matrix, the tendency of NS to absorb water results in an incomplete hydration reaction [20,84,85]. For mixes with 0.45% MWCNTs, the appearance of the highest strength was postponed to an NS content of 0.6%. This can be attributed to the fact that increasing MWCNTs provide more seeding surfaces, promoting the reaction between NS and CH during the hydration process.

### 3.2. Compressive Strength

**Figure 5:**
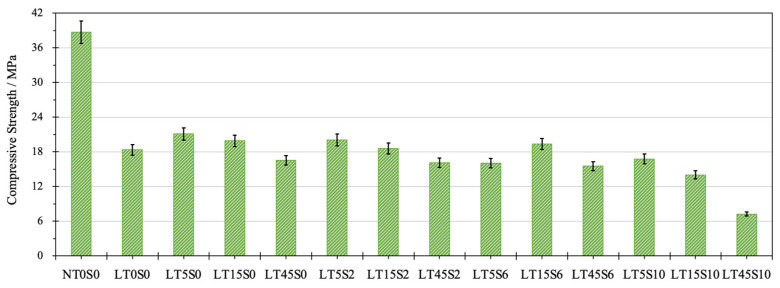
Compressive strength of cement mortar.

The compressive strength of cement mortar is shown in Figure 5. Without the addition of nano additives, the 28-day compressive strength of LWCM mix LT0S0 was 17.97 MPa, which reached 47% of that of NACM (38.72 MPa), with 1.06% strength loss per 1% density reduction. After the addition of nano additives, the positive effects were measured for mixes LT5S0, LT15S0, LT15S2, and LT15S6, and a negative influence was observed in mixes LT45S0, LT5S6, LT5S10, LT15S10, LT45S2, LT45S6, and LT45S10. In contrast with LT0S0, mix LT5S0, modified by 0.05% of MWCNTs without the presence of NS, showed the most impressive performance in compressive strength, which increased by 14.92% to 21.10 MPa. The strength reached 54.49% of the strength of NACM, and its density was only 58.05% of the NACM density. Mix LT45S10 accounted for the lowest compressive strength, decreasing by 60.47% to 7.26 MPa. This can be attributed to the increased amount of nano additives, leading to agglomeration, generating weak zones in the cement matrix and increasing the difficulty of dispersion [83,86,87]. This also explains why mixes with either 0.45% MWCNTs or 1.0% NS all demonstrated different extents of drops in strength.

When the content of NS was fixed at 0.06%, along with the increment of MWCNT dosage, the strength value first increased and then declined, peaking at 0.15% of MWCNTs with a value of 19.48 MPa. Meanwhile, at the constant addition of NS at 0.2 and 1.0%, when the content of MWCNTs was enhanced, the compressive strength achieved the highest value at 0.05% MWCNTs. Afterwards, the strength exhibited a continuous reduction by 21.72, 20.60, and 16.38%, respectively, when the dosage of MWCNTs reached 45%. This result is in accordance with some studies [81,88], which reported that the optimal dosage of MWCNTs for improving the compressive strength of cement-based materials existed below 0.1% by cement mass. When MWCNT content crosses the beneficial threshold, they can compete with OPC particles and FACs for the superplasticizer, adversely impacting the strength growth [88]. At the constant amount of MWCNTs at 0.05, 0.15, and 0.45%, along with the growth of NS content from 0% to 1.0%, the compressive strength of each LWCM all experienced different degrees of fluctuation. When NS content reached 1.0%, the lowest strength values were observed. Besides the fact that NS competes with OPC for water, reducing the hydration degree, the strength loss can also be connected with the poorly dispersed NS particles and the lack of CH to further react with the unconsumed NS [89].

### 3.3. Environmental and Economic Assessment

#### 3.3.1. Environmental Impacts and Interpretation of Lifecycle Assessment

**Figure 6:**
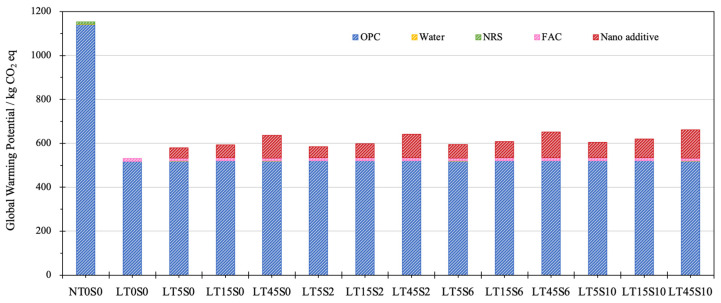
Greenhouse gas emission of cement mortar samples.

The values of GWP for different mixes are shown in Figure 6. The greenhouse gas emission of nano additives consists of the CO_2_ generated due to MWCNTs, NS, surfactant Melment F10, and the ultrasonication treatment for nano suspension. The CO_2_ emission in the lifecycle of cement mortars elementally came from the production of cement as well as nano additives. Fine aggregates—both NRS and FACs—contributed to a very limited emission of greenhouse gas. NACM possessed greater total GWP, with approximately 1200 kg CO_2_ eq, while the GWP values of LWCM ranged from 550 to 690 kg CO_2_ eq. In contrast with NT0S0, the utilization of FACs led to a 53.68% decline of GWP for mix LT0S0. This demonstrates that the utilization of FACs to manufacture LWCM can significantly reduce environmental threats.

The use of nano additives enhanced the greenhouse gas emissions, with the greatest GWP value of modified LWCM recorded for LT45S10, displaying an increment of 23.83% to 685.86 kg CO_2_ eq, whereas mix LT5S0 possessed the lowest growth of GWP by 8.84% to 603.44 kg CO_2_ eq. Along with the growth of MWCNT content from 0.05% to 0.45%, at the constant addition of NS, the greenhouse gas emission exhibited a significant uptrend by an average of 9.22%. At the constant dosage of MWCNTs, the binary addition of NS from 0.0% to 1.0% accounted for a slight enhancement of GWP by 4.19% on average.

**Figure 7:**
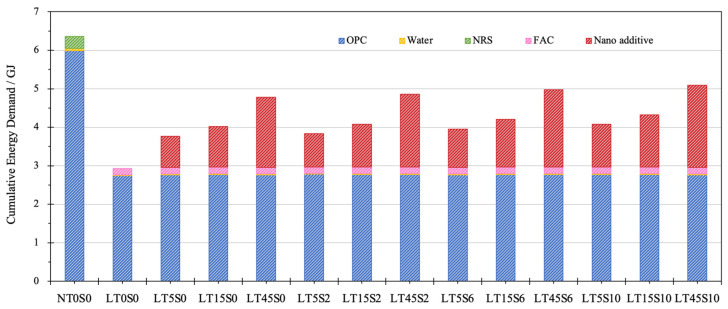
Energy consumption of cement mortar samples.

Figure 7 displays the comparison of the CED of different cement mortars. Similar to GWP, the energy consumption of nano additives is composed of the production of MWCNTs, NS, surfactant, and ultra-sonication. It can be noticed that the energy consumption of cement mortar was mainly attributed to OPC, which occupied more than half of the energy demand for all the mixes. The production and processing of NRS and FACs accounted for the very scarce energy demand. Another highly energy-intensive procedure belonged to the fabrication and treatment of nano additives. Especially for mix LT45S10, the energy requirement of nano additives almost took up the same proportion as that of OPC.

Similar to the results for GWP, mix NT0S0 with NRS exhibited the highest energy requirement of 7 GJ, whereas the CED of LWCM varied between 3.2 and 5.5 GJ. Without the addition of nano additives, the CED of LT0S0 (3.24 GJ) was 53.32% less than that of NACM, testifying to the fact that using waste LWA FACs can effectively conserve energy. The incorporation of nano additives led to a remarkable enhancement in the CED, and the same increasing evolution as GWP was observed when the amount of MWCNTs and NS grew. In contrast to mix LT0S0, the most considerable increment in CED was recorded for mix LT45S10, with a 67.47% increase to 5.43 GJ, while specimen C5S0 had the least growth in energy consumption by 26.73%. Compared with the mixes containing only CNTs, the binary usage of NS brought very limited improvement in CED.

#### 3.3.2. Economic Analysis

**Figure 8:**
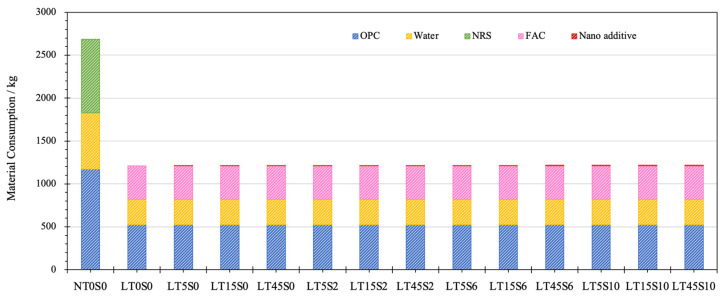
Material consumption of cement mortar samples.

In terms of TMC, as shown in Figure 8, the material consumption mainly came from OPC, water, and fine aggregates. Incorporating nano additives barely influenced the total values of material consumption. It can be noticed that, at the same w/c ratio and fine aggregate/OPC ratio, the usage of industrial waste FACs contributed notably to the conservation of raw materials, with only 45.00% of the raw materials required to fabricate LWCM (mix LT0S0), in contrast with the consumption to produce NACM (1700 kg). Even for the mix encompassing the highest amount of MWCNTs and NS, mix LT45S10 only demanded 1219.68 kg of raw materials for manufacturing one cubic meter of cement mortar, which was 71.75% of the material consumption used to produce NACM.

**Figure 9:**
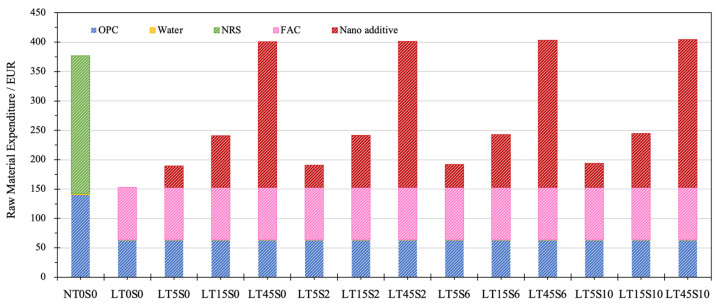
Total material cost of cement mortar samples.

Figure 9 displays the outcomes of RME. The material costs of NACM elementally came from OPC and NRS, while the major expenditure of LWCM was composed of the inputs in OPC, FACs, and nano additives. It is worth mentioning that the costs of transportation, sonication treatment, and mortar processing were excluded from the calculation. The costs of nano additives consist of the expenditure of MWCNTs, NS, and surfactants. Comparing mixes NT0S0 and LT0S0, the RME of the latter was 59.41% less than that of the former, demonstrating that FACs can significantly economize the production cost, which primarily benefits from the decreased material consumption of OPC and fine aggregates. Besides the apparent direct economic benefits, the indirect advantages due to declining fees in landfill and waste processing are also promising [31]. According to Martinez-Lage et al. [90], incorporating waste aggregates into fabricating concrete materials can contribute to 35% of the reduction in waste management and 50% of the conservation of natural resources. Because FACs are a waste material from industry, it is prospective that their price may decline in the future, which can further improve the economic viability of LWCM [24].

The addition of nano additives greatly improved the total material costs of LWCM, fundamentally owing to the expensive material price of MWCNTs. The greatest RME required to produce cement mortar was observed for mixes containing 0.45% of MWCNTs, i.e., LT45S0, LT45S2, LT45S6, and LT45S10, with costs of EUR 400.62, 401.39, 402.93, and 404.47 per cubic meter, respectively. The expenditure to generate one cubic meter of NACM was EUR 376.81, which was 7.43% less than that of LT45S10, indicating, due to the expensive price of MWCNTs, the inclusion of MWCNTs at higher dosages is of low economic viability for mass application in engineering practice. The inclusion of NS, however, scarcely affected the total material costs. At the fixed amount of MWCNTs, the further addition of NS, with the increment of its content from 0.0% to 1.0%, only contributed to an average increase of 1.53% in RME.

#### 3.3.3. Effects of Nano Additives on Environmental and Economic Feasibility

**Figure 10:**
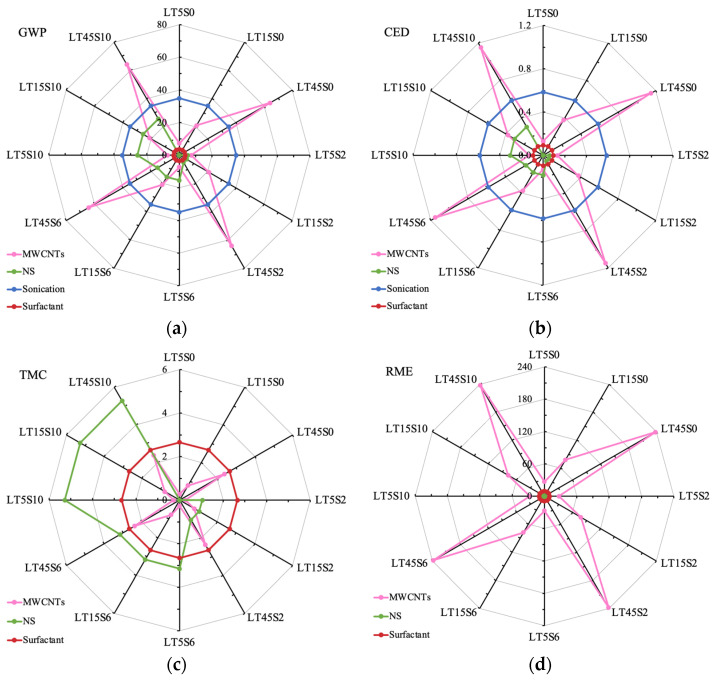
Environmental and economic impacts of nano additives. (**a**) Global warming potential (kg CO_2_ eq/m^3^). (**b**) Cumulative energy demand (GJ/m^3^). (**c**) Total material consumption (kg/m^3^). (**d**) Raw material expenditure (EUR/m^3^).

To identify the influence of nano additives on the environmental and economic impacts of LWCM, in Figure 10 the CO_2_ emission, energy demand, material consumption, and costs of MWCNTs, NS, and surfactant, as well as sonication treatment, were calculated. In Figure 10a,b, the same evolution patterns were observed for greenhouse gas emissions and energy consumption. The GWP of nano additives varied from 45 to 128 kg CO_2_ eq per cubic meter of mortar, and the CED changed between 0.8 and 2.1 GJ. Using surfactant contributed to the least amount of CO_2_ generation and energy requirement, with a constant of 3.65 kg CO_2_ eq and 0.09 GJ, respectively. For mixes with 0.45% of MWCNTs, most of the greenhouse gases and energy demand were attributed to MWCNTs, followed by the ultra-sonication treatment and then the addition of NS, whereas, for mixes containing 0.05 and 0.15% of MWCNTs, the process of ultra-sonication (34.9 kg CO_2_ eq and 0.59 GJ) accounted for the highest proportion of GWP and CED. It is worth noticing that the growth in MWCNT content led to a sharp increase in both GWP and CED values, and with the increment of NS dosage a mild enhancement was observed. Even at the amount of 1.0% inclusion, GWP and CED ascribed to NS did not surpass those resulting from the sonication process.

Concerning material consumption, approximately 2.93 to 10.31 kg of raw materials were used in the section on nano additives. As can be seen in Figure 10c, MWCNTs occupied the least proportion for all the modified LWCM mixes. When the content of NS was 0.6 and 1.0%, the highest nano additive consumption came from NS, while at the amount of 0.0 and 0.2%, the consumption was primarily due to the surfactant, which was fixed at 2.66 kg. In Figure 10d, the costs of NS and surfactant remained at low values, with an average of 2.31 EUR for the former and a constant 9.68 EUR for the latter. The total costs of MWCNTs varied from EUR 27 to 238, and as the content of MWCNTs grew the expenditure to include MWCNTs showed a sharp increase. The interaction of NS with MWCNTs allowed a decrease in the amount of MWCNTs consumed and thus led to the reduction of production costs, especially when a similar mechanical strength was achieved. For mixes LT45S0 and LT5S6, the flexural strength was similar (1.29 and 1.23 MPa, respectively); however, by adding 0.6% NS, the material cost decreased by 52.1%.

### 3.4. Multi-Criteria Assessment and Optimization

#### 3.4.1. Sustainability Index and Economic Index

As discussed above, apparently, no obvious pattern was observed for the evolution of environmental and economic impacts along with the changes in mechanical strength. The most remarkable compressive strength and flexural strength were achieved by mixes LT5S0 and LT15S2, respectively. The overall material consumption of modified LWCM was solely determined by the material demand in cement and fine aggregates and showed very few changes with the addition of MWCNT and NS dosages. Energy consumption, production cost, and greenhouse gas emissions displayed similar tendencies with the variation of nano additive dosages and were considerably affected by the quantity of MWCNTs. Thus, to systematically compare different mixes and identify the optimal LWCM mix, the five selected mixes that achieved positive results in both flexural and compressive strength tests were evaluated based on GWP, CED, RME, and cumulative strength, which was the sum of flexural and compressive strength.

**Figure 11:**
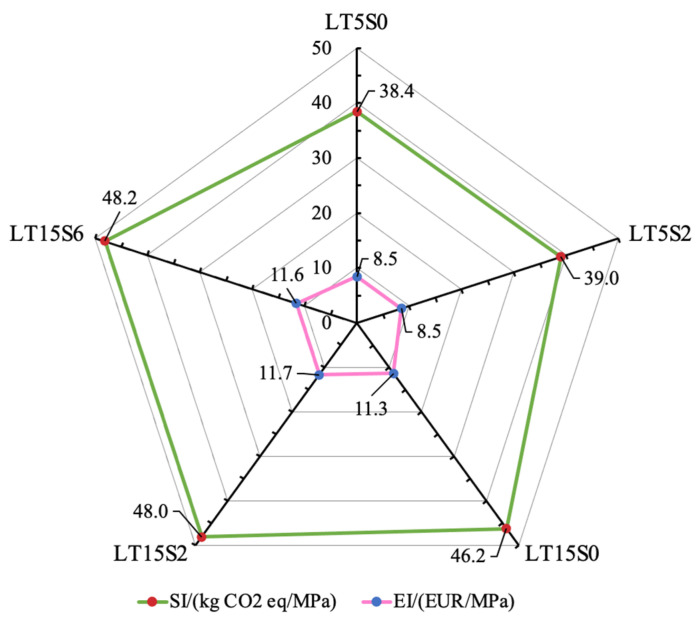
Sustainable and economic index of LWCM.

SI and EI were calculated and are presented in Figure 11. Based on the production of per cubic meter mortar, SI is assessed via the CO_2_ emission per MPa strength, whereas EI signifies the manufacturing cost per MPa strength. Note that the lower the values of SI and EI, the better environmental sustainability and economic viability are achieved. The highest SI and EI values were obtained by mixes LT15S6 (48.2 kg CO_2_ eq/MPa) and LT15S2 (11.7 EUR/MPa), respectively. This can be attributed to the greater amount of greenhouse gas yielded for mix LT15S6, as well as the lower total strength value of mix LT15S2, even if it possessed the most remarkable flexural characteristics. This demonstrates that, even if a mix achieved optimal performance in one aspect, its comprehensive behavior may not surpass the other mixes. The lowest EI and SI were both seen for mix LT5S0, the value of which was 8.5 EUR/MPa and 38.4 kg CO_2_ eq/MPa, respectively. Mix LT5S2 showed a similar sustainability and economic index to LT5S0, with only 1.46% and 0.59% differences in SI and EI values, respectively. However, its flexural strength was 1.2 times more than that of LT5S0, demonstrating that, with the significance attached to the improvement of flexural behaviors, LT5S2 could be a better mix than LT5S0. While considering the best compressive performance obtained by mix LT5S0, it could be a more optimal mix when the compressive strength was the primary factor. Especially in some developing countries, concrete is usually selected based on the achieved compressive strength [35].

#### 3.4.2. TOPSIS Optimization

Five LWCM mixes (LT5S0, LT5S2, LT15S0, LT15S2, and LT15S6), modified by both MWCNTs and NS, were subjected to TOPSIS analysis to decide the optimal mix. Six criteria, in terms of mechanical properties (compressive strength, fc, and flexural strength, ff), environmental sustainability (GWP and CED), and economic feasibility (TMC and RME), were adopted to evaluate the comprehensive behaviors of the five alternatives. Based on the procedures shown in Figure 3 and the values of each parameter, at first, a decision matrix, D, as defined in Equation (3), was constructed by combining the alternatives and criteria. Afterwards, the decision matrix was normalized and multiplied by the corresponding weight of each parameter in Table 7 to generate the weighted normalized decision matrix, N, for different circumstances. In Equation (4), the normalized matrix, N1, for the first scenario was displayed. Then, the alternatives were idealized to obtain the positive and negative solutions, S^+^ and S^−^, in Equations (5) and (6), respectively. In this study, the best LWCM mix belonged to the one with the highest compressive and flexural strength, as well as the lowest values of GWP, CED, TMC, and RME. In Equations (7) and (8), the difference between each alternative and the best or worst solutions was calculated, respectively, and in Equation (9) the RCC values of each alternative for the first circumstance was calculated. The maximal value of RCC signified the optimal LWCM mix with the best comprehensive performance.

(3) $$D=\left[\begin{array}{cccccc} 21.10 & 1.31 & 629.56 & 4.62 & 1212.30 & 189.62\\ 20.80 & 1.57 & 636.48 & 4.70 & 1213.35 & 190.39\\ 19.92 & 1.42 & 693.76 & 5.85 & 1212.81 & 240.62\\ 18.58 & 2.14 & 699.28 & 5.92 & 1213.86 & 241.39\\ 19.37 & 1.60 & 708.96 & 6.03 & 1215.97 & 242.93 \end{array}\right]$$

where:

The row follows the order of criterion *fc* (MPa), *ff* (MPa), GWP (kg CO_2_ eq/m^3^), CED (GJ/m^3^), TMC (kg/m^3^), and RME (EUR/m^3^), while the column follows the sequence of mixes LT5S0, LT5S2, LT15S0, LT15S2, and LT15S6.

(4) $$N_{1}=\left[\begin{array}{cccccc} 0.0787 & 0.0597 & 0.0696 & 0.0631 & 0.0745 & 0.0635\\ 0.0776 & 0.0718 & 0.0703 & 0.0642 & 0.0745 & 0.0638\\ 0.0743 & 0.0648 & 0.0767 & 0.0799 & 0.0745 & 0.0806\\ 0.0693 & 0.0976 & 0.0773 & 0.0808 & 0.0745 & 0.0809\\ 0.0723 & 0.0731 & 0.0784 & 0.0823 & 0.0747 & 0.0814 \end{array}\right]$$

(5) $$S_{1}^{+}=\left[\begin{array}{ccc} 0.0787 & 0.0976 & \begin{array}{ccc} 0.0696 & 0.0631 & \begin{array}{cc} 0.0745 & 0.0635 \end{array} \end{array} \end{array}\right]$$

(6) $$S_{1}^{-}=\left[\begin{array}{ccc} 0.0693 & 0.0597 & \begin{array}{ccc} 0.0784 & 0.0823 & \begin{array}{cc} 0.0747 & 0.0814 \end{array} \end{array} \end{array}\right]$$

(7) $$R_{1}^{+}={\left[\begin{array}{ccc} 0.0379 & 0.0259 & \begin{array}{ccc} 0.0415 & 0.0276 & 0.0375 \end{array} \end{array}\right]}^{T}$$

(8) $$R_{1}^{-}={\left[\begin{array}{ccc} 0.0293 & 0.0303 & \begin{array}{ccc} 0.0078 & 0.0379 & 0.0137 \end{array} \end{array}\right]}^{T}$$

(9) $${RCC}_{1}={\left[\begin{array}{ccc} 0.44 & 0.54 & \begin{array}{ccc} 0.16 & 0.58 & 0.27 \end{array} \end{array}\right]}^{T}$$

Following the same steps, the RCC values in the other three situations with different weighting factors for the criteria were concluded in Equations (10)–(12), respectively.

(10) $${RCC}_{2}={\left[\begin{array}{ccc} 0.26 & 0.40 & \begin{array}{ccc} 0.17 & 0.75 & 0.34 \end{array} \end{array}\right]}^{T}$$

(11) $${RCC}_{3}={\left[\begin{array}{ccc} 0.64 & 0.71 & \begin{array}{ccc} 0.15 & 0.39 & 0.16 \end{array} \end{array}\right]}^{T}$$

(12) $${RCC}_{4}={\left[\begin{array}{ccc} 0.61 & 0.69 & \begin{array}{ccc} 0.11 & 0.40 & 0.18 \end{array} \end{array}\right]}^{T}$$

**Figure 12:**
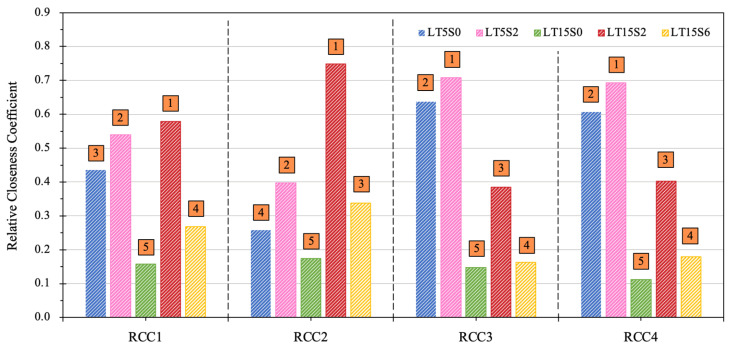
TOPSIS analysis of LWCM mixes (the sequence of the mix rating was marked from 1 to 5 for each circumstance).

Figure 12 shows the results of the TOPSIS analysis under the four scenarios. As can be observed, the optimal LWCM mix was consistently observed for mixes LT15S2 or LT5S2. This differs from the outcomes in the multi-response assessment via SI and EI, in which mix LT5S0 acquired the lowest SI and EI values. The distinguishment occurring in the evaluation can be related to the assessment procedures. When SI and EI were calculated, normalization was excluded in the process and the criteria of flexural as well as compressive strength were included via simple cumulation. However, considering the fact that the compressive strength was nearly ten times more than the flexural strength for each mix, the advantages of mix LT15S2, which possessed the highest flexural strength, were underestimated, whereas the significance of compressive behavior was magnified. Hence, under the circumstance where compressive strength took priority, the mix with the highest strength value achieved the best ranking in the assessment. It is noticeable that, under all four conditions, mix LT15S0, with only 0.15% of CNTs and no NS, had the lowest RCC value, indicating that it was the most negative choice among LWCM mixes. This can be ascribed to its high environmental influence and production cost, as well as its mediocre mechanical behaviors.

It can be seen in Figure 12 that, when the five criteria were assigned the same significance (in the first optimization situation), the best LWCM mix belonged to LT15S2. The second-ranking priority was recorded for mix LT5S2. In the second circumstance, it is worth noticing that, when the mechanical properties had a higher weight among the six criteria, mix LT15S2 still obtained the most positive result, indicating that, although it failed to achieve the highest compressive strength, it still showed the best mechanical behavior given a comprehensive evaluation. Meanwhile, mix LT5S0 only ranked in the fourth sequence, despite it having the highest compressive strength, illustrating its inferior mechanical performance from a comprehensive perspective. In the third and fourth scenarios, where environmental and economic viability were attached with greater weights, respectively, a similar trend was witnessed that the peak ranking belonged to mix LT5S2, followed by LT5S0. While mixes incorporating 0.15% of MWCNTs all exhibited comparatively smaller RCC, owing to their increasing inclusion of MWCNTs, which tremendously improved the greenhouse gas emissions and energy demand, they required larger economic investment for fabrication. The preference order of LWCM mixes under different conditions is concluded below in Equations (13)–(16), respectively:

(13) C15S2>C5S2>C5S0>C15S6>C15S0

(14) C15S2>C5S2>C15S6>C5S0>C15S0

(15) C5S2>C5S0>C15S2>C15S6>C15S0

(16) C5S2>C5S0>C15S2>C15S6>C15S0

Overall, the priority sequence of LWCM mixes varied according to the weighting factors as well as the optimization requirements in different circumstances. With equal significance attached to all the criteria, mix LT15S2 comprehensively outperformed the other mixes. Similarly, it occupied the priority ranking position if mechanical behaviors were assumed to be more important. When the environmental sustainability and economic feasibility were preferentially evaluated, mix LT5S2 was the optimal choice. This indicates that the inclusion of MWCNTs and NS binarily contributed to improved performance given a thorough comprehensive evaluation of the mixes, in contrast to those with only the single addition of MWCNTs. Especially when the mechanical criteria were given more significance, all three mixes binarily modified by both MWCNTs and NS outperformed those encompassing only MWCNTs. Considering that mix LT5S2 exhibited impressive ranking in all four situations with the first or the second priority, combining its remarkable SI and EI values, it was recommended in this study as the optimal LWCM mix.

## 4. Conclusions

This study produced a green LWCM with FACs as fine aggregates and MWCNTs and NS as modifiers to provide synergic effects on the mechanical behaviors. The sustainability and economic feasibility were assessed via indicators of CO_2_ emissions, energy demand, material consumption, and cost within the confines of cradle-to-gate assessment. Engaged with the outcomes from mechanical strength tests, a multi-criteria optimization was conducted by SI, EI, and the TOPSIS method to determine the optimal mix. LWCM is a potential sustainable material for producing lightweight cement concrete and blocks for building and construction components. According to the acquired results, primary conclusions can be derived as follows:

The addition of nano additives contributed to greater flexural behaviors, and the most remarkable flexural strength was measured for mix LT15S2, which contained 0.15% of MWCNTs as well as 0.2% of NS, with an 89.38% increment observed to 2.14 MPa. Both the positive and negative effects of nano additives were observed for the compressive strength, and the highest strength value belonged to mix LT5S0, which incorporated 0.05% of MWCNTs and no NS. The compressive strength was increased by 14.92% to 21.10 MPa. Compared to the compressive behavior, more outstanding modifying effects by nano additives were observed on flexural performance. The reason for this can be associated with the remarkable tensile strength of MWCNTs and their effects on bridging micro-cracks.

Compared to NACM, the utilization of recycled lightweight aggregate FACs decreased the environmental impacts and improved the economic viability. The incorporation of nano additives led to the enhancement of GWP, CED, and RME. In particular, due to the high price and intensive energy demand of MWCNTs, lower dosages of MWCNTs at 0.05% and 0.15% were more sustainable. The binary use of NS benefited lower production costs and improved sustainability.

The lowest SI and EI were both reported for mix LT5S0. Mix LT5S2, incorporated with 0.05% MWCNTs and 0.2% NS, exhibited the second smallest values, but given its higher flexural strength and similar compressive strength compared with LT5S0, it could be an alternative choice for the optimal mix.

The analysis of TOPSIS indicates that, pertaining to comprehensive behaviors, mixes binarily modified by MWCNTs and NS outperformed those with the single addition of MWCNTs. When the six criteria had the same significance or the mechanical properties possessed higher weights, the optimal mix belonged to LT15S2, followed by mix LT5S2. Meanwhile, in the situation where environmental or economic parameters were assigned greater importance, the optimal mix was achieved by LT5S2.

The different results acquired in TOPSIS analysis can be linked to the lack of normalization procedure during the calculation of the SI and EI values, which minimized the influence of flexural strength and magnified the effects of compressive strength. Based on the multi-parameter assessment, the optimal mix in this study was recommended to be LT5S2.

This study provided a preliminary reference to the eco-efficiency analysis on lightweight cement-based materials. Notwithstanding, there are still some limitations worth mentioning. The FU of the LCA was set as 1 cubic meter of mortar, which is not feasible enough when the concrete grades and the material function during construction practice need to be considered. Besides the cradle-to-gate analysis exhibited in this study, a full-scale LCA is favorable, taking into consideration the recycling or disposal stage in order to understand the possibility of material valorization at the end-life. Meanwhile, the optimization only involved mechanical strength as the criteria for engineering properties, and parameters related to durability were lacking. For future studies, it is recommended to include the end-life-stage analysis into LCA and combine strength parameters with durability in the optimization. To promote the application of the material, a comparison of the lifecycle to traditional cement-based materials considering engineering practices is suggested.

## Figures and Tables

**Table 1:** Material properties of OPC and FACs in the experiments.

Material Properties	OPC	FACs
Appearance	Grey powder	White or grey powder
Initial setting time (min)	>60	-
Loss on ignition (%)	<5.0	-
Sulphate content (%)	<3.5	-
Chloride content (%)	<0.1	-
Bulk density (g/cm^3^)	0.9–1.5	0.37–0.40
True density (g/cm^3^)	-	0.6–0.7
pH value	-	7–8
Thermal conductivity (w/mk)	-	0.036–0.060
Early compressive strength (2 days) (MPa)	>10.0	-
Standard compressive strength (28 days) (MPa)	42.5–62.5	20–40

**Table 2:** Mix design and material consumption for 1 m^3^ cement mortar.

Mixture	OPC (kg)	Water (kg)	Melment F10 (kg)	Fine Aggregates (kg)		MWCNTs		NS	
				NRS	FACs	%	kg	%	kg
NACM									
NT0S0	1171.88	656.25	-	859.34	-	-	-	-	-
LWCM									
LT0S0	527.34	295.31	-	-	386.72	-	-	-	-
Modified LWCM									
LT5S0	527.34	295.31	2.66	-	386.72	0.05	0.27	-	-
LT15S0						0.15	0.78	-	-
LT45S0						0.45	2.38	-	-
LT5S2						0.05	0.27	0.2	1.05
LT15S2						0.15	0.78	0.2	1.05
LT45S2						0.45	2.38	0.2	1.05
LT5S6						0.05	0.27	0.6	3.16
LT15S6						0.15	0.78	0.6	3.16
LT45S6						0.45	2.38	0.6	3.16
LT5S10						0.05	0.27	1.0	5.27
LT15S10						0.15	0.78	1.0	5.27

**Table 3:** LCI from Ecoinvent 3.0 database.

Inventory	Ecoinvent 3.0 Database
OPC	Cement, Portland {RoW} | market for | Alloc Def, U
NRS	Sand {Glo} | market for | Alloc, Def, U
Water	Water, deionized, from tap water, at user {Row} | market for | Alloc Def, U
Melment F10	Plasticiser, for concrete, based on sulfonated melamine formaldehyde {Glo}| market for | Alloc Def, U

**Table 4:** Categories and methods of impact assessment in LCA.

Impact Category	Abbreviation	Method	Unit
Global warming potential	GWP	IPCC GWP 100a	kg CO_2_ eq
Cumulative energy demand	CED	Cumulative Energy Demand	GJ

**Table 5:** Material price (unit: EUR/kg).

OPC	Water	FACs	NRS	Melment F10	MWCNTs	NS
0.12 ^1^	0.002 ^2^	0.23 ^3^	0.27 ^4^	3.64 ^5^	100 ^6^	0.73 ^7^

^1^https://schwenk.lv, accessed on 26 August 2023; ^2^ The price is based on the water price in Latvia; ^3, 4, 6, 7^ https://www.alibaba.com/, accessed on 26 August 2023; ^5^ https://www.basf.com/global/en.html, accessed on 26 August 2023.

**Table 6:** Indicators and methods of economic analysis.

Impact Category	Abbreviation	Method	Unit
Total material consumption	TMC	Direct analysis	kg
Raw material expenditure	RME	Direct analysis	EUR

**Table 7:** Weighting factors in TOPSIS analysis.

Circumstance	Mechanical Criteria		Environmental Criteria		Economic Criteria	
	f_c_	f_f_	GWP	CED	TMC	RME
1	0.17	0.17	0.17	0.17	0.17	0.17
2	0.3	0.3	0.1	0.1	0.1	0.1
3	0.1	0.1	0.3	0.3	0.1	0.1
4	0.1	0.1	0.1	0.1	0.3	0.3

## Data Availability

The original contributions presented in the study are included in the article.
